# Misleading Procalcitonin in Patients With Staphylococcus aureus Bacteremia: A Report of Two Cases

**DOI:** 10.7759/cureus.43415

**Published:** 2023-08-13

**Authors:** Sharath Kommu, Vidyasagar Cirra

**Affiliations:** 1 Hospital Medicine, Marshfield Clinic, Rice Lake, USA

**Keywords:** mssa, mrsa, staphylococcus aureus, bacteremia, false-negative procalcitonin, pct, procalcitonin

## Abstract

Procalcitonin (PCT) is an important biomarker for bacterial infection with a high negative predictive value. It is almost always positive in patients who are bacteremic with pathogenic bacteria. Here, we report two cases of *Staphylococcus aureus* bacteremia, where PCT levels were unexpectedly negative. This uncommon occurrence challenges the assumption of PCT’s infallibility as a diagnostic marker in patients with true bacteremia. The first case is a 55-year-old woman with no past medical issues who presented with one week of generalized weakness and two days of fever and chills. Though her white blood cell (WBC) count and c-reactive protein (CRP) were elevated, PCT was normal, with no apparent source of infection, and hence antibiotic differed. However, her blood cultures returned positive for methicillin-resistant *Staphylococcus aureus* (MRSA). The patient was started on vancomycin and discharged on daptomycin, she responded appropriately and improved. The second case is an intravenous (IV) drug user, a 40-year-old woman, who presented with septic arthritis and osteomyelitis involving the right hip. She had blood cultures positive for methicillin-susceptible *Staphylococcus aureus* (MSSA); however, a PCT check on the day of positive blood cultures and various occasions subsequently was normal. These two cases remind us that we cannot over-rely on one test to rule out bacterial infection and should consider the whole clinical picture. They highlight the need for vigilance among clinicians that PCT can rarely be negative in cases of true bacteremia in spite of its high negative predictive value. Physicians and antibiotic stewardship programs should be cautious and aware of this potential pitfall when utilizing PCT as a diagnostic tool.

## Introduction

While several biomarkers exist for detecting infection such as white blood cell (WBC) count, c-reactive protein (CRP), and erythrocyte sedimentation rate (ESR), procalcitonin (PCT) has garnered significant attention in the past decade due to its high negative predictive value for bacterial infections. Consequently, PCT has emerged as a valuable tool in antibiotic stewardship programs, aiding in the reduction of inadvertent antibiotic administration [1]. Its sensitivity further improves with a decreasing threshold value [2,3]. The cases of PCT being negative in true bacteremia are exceedingly rare [4,5]. Here, we present two cases of true bacteremia with negative PCT. These cases highlight that relying on PCT-guided therapy in cases of suspected bacterial infections may be a misguided step on certain occasions.

Case 1 of this study was previously presented as a poster at the Society of Hospital Medicine (SHM) Converge 2023 Conference in Austin, TX.

## Case presentation

Case 1

A 55-year-old woman with no known past medical issues presented to the emergency department (ED) with a one-week history of generalized weakness, and fevers and chills for two days. Workup showed results as indicated in Table 1. There was no evidence of skin infection or urinary symptoms to suggest a urinary tract infection (UTI). The viral tests for coronavirus disease 2019 (COVID-19), influenza, and respiratory syncytial virus (RSV) returned negative. Since PCT was negative (less than 0.05 ng/mL) and there was no apparent source of infection, antibiotics were not initiated. Subsequently, two blood cultures returned positive for methicillin-resistant *Staphylococcus aureus *(MRSA). A repeat PCT also returned negative. The patient exhibited no focal symptoms or signs to suggest a source of infection. Given the presence of MRSA bacteremia, the patient had an extensive evaluation to find the source of the infection. Initially, there was a dilemma of potential mitral valve vegetation on transthoracic echocardiogram (TTE); however, the confirmatory test of transesophageal echocardiogram (TEE) ruled out any vegetation. In addition, computer-aided tomography (CT) scans of the chest, abdomen, and pelvis also did not reveal any potential source of infection.

**Table 1:** Blood work results of the patient in Case 1. WBC: white blood cell; CRP: c-reactive protein; PCT: procalcitonin

Parameter	Result	Normal range
WBC count	15.6x10^3^/µL	4.1-10.9x10^3^/µL
Hemoglobin	12.1 g/dL	11.7-15.5 g/dL
Platelet count	370x10^3^/µL	150-450x10^3^/µL
CRP	14.3 mg/dL	0.0-1.0 mg/dL
PCT	<0.05 ng/mL	0.0-0.1 ng/mL

An infectious disease specialist was consulted and the patient was appropriately treated with intravenous (IV) vancomycin during her hospital stay and discharged on IV daptomycin to complete a four-week antibiotic course. The infectious disease specialist opted to treat it as a complicated bacteremia without endocarditis and hence the four-week antibiotic course was chosen.

Case 2

A 40-year-old woman with polysubstance use disorder, including IV drug abuse, presented to the ED with two months of back and right hip pain. The pain worsened alongside difficulty with ambulation and the development of bedsores. She denies having fevers or chills. At the time of admission, her heart rate was 110 beats/min, her blood pressure was 140/65 mmHg, and her temperature was 36.5°C. Blood work is shown in Table 2.

**Table 2:** Blood work results of the patient in Case 2. WBC: white blood cell; ESR: erythrocyte sedimentation rate; CRP: c-reactive protein; PCT: procalcitonin

Parameter	Result	Normal range
WBC count	10.8x10^3^/µL	4.1-10.9x10^3^/µL
Hemoglobin	10.6 g/dL	11.7-15.5 g/dL
Platelet	400x10^3^/µL	150-450x10^3^/µL
ESR	122 mm/h	0-17 mm/h
CRP	9.7 mg/dL	0.0-1.0 mg/dL
PCT	<0.05 ng/mL	0.0-0.1 ng/mL

**Figure 1:**
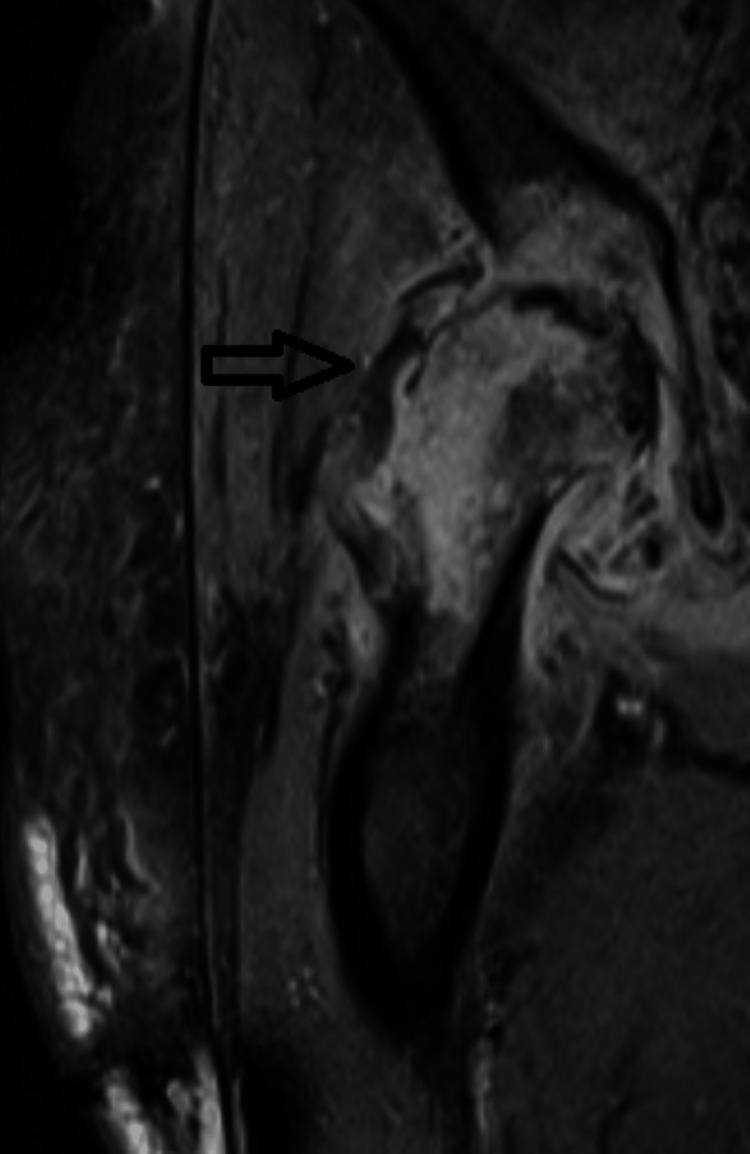
MRI T1-weighted fat saturation post-contrast coronal image of right hip showing sequelae of septic arthritis and extensive osteomyelitis (arrow).

One of the two blood cultures was positive for methicillin-sensitive *Staphylococcus aureus* (MSSA). Joint fluid aspirate from the right hip also showed MSSA. PCT checked various times during this hospitalization was negative (<0.05 ng/mL). A CT scan of the pelvis showed a mottled appearance of the right femoral head and right acetabulum with soft tissue infiltration surrounding the right hip joint, with findings concerning septic arthritis. Magnetic resonance imaging (MRI) of the right hip shows marked abnormality compatible with sequelae of septic arthritis and extensive osteomyelitis, including full-thickness cartilage destruction of the right hip joint with bone loss from the acetabular dome and superior femoral head (Figure 1). Orthopedic surgery and infectious disease specialists evaluated the patient. She underwent right hip resection arthroplasty (Girdlestone) and placement of resorbable antibiotic beads. Infectious disease recommended six weeks of IV cefazolin, and the patient was discharged to a nursing home for continued care.

This is a typical case of septic arthritis secondary to IV drug abuse; however, despite positive blood cultures with MSSA, when PCT was checked on various occasions during the hospital stay, it returned negative.

## Discussion

PCT is a 116-amino acid residue first explained by Le Moullec et al. in 1984 [6]. Its diagnostic significance; however, was not recognized until 1993, when Assicot et al. demonstrated a positive correlation between high serum levels of PCT and patients with positive findings for bacterial infection and sepsis (e.g., positive blood cultures) [7,8]. Without systemic inflammation, PCT synthesis is restricted to thyroid neuroendocrine cells, and the protein is not released into the blood until it cleaves into its mature form, calcitonin [9-12]. Thus, serum PCT is typically undetectable in healthy persons [11]. The extra-thyroid synthesis of PCT has been found to occur in the liver, pancreas, kidney, lung, and intestine, and within leukocytes, where the synthesis of PCT is suppressed [8]. However, in bacterial infections, PCT synthesis is induced in these tissues, which is later released into the bloodstream. PCT serum levels can also become elevated among patients with noninfectious conditions, such as trauma, burns, carcinomas (medullary C cell, small cell lung, and bronchial carcinoid), immunomodulatory therapy that increases proinflammatory cytokines, cardiogenic shock, the first two days of a neonate’s life, during peritoneal dialysis treatment, and in cirrhotic patients (Child-Pugh Class C) [13-15]. PCT levels are also elevated in patients suffering from various degrees of chronic kidney disease [16].

Antimicrobial stewardship programs play a critical role in promoting the judicious and appropriate utilization of antibiotics, aiming to mitigate adverse effects and combat antibiotic resistance. In contrast to conventional nonspecific inflammatory markers like erythrocyte sedimentation rate (ESR) and c-reactive protein (CRP), procalcitonin (PCT) has emerged as a valuable tool for identifying bacterial infections due to its high specificity [8]. As a result, PCT is increasingly integrated into antibiotic stewardship programs as a reliable indicator to guide decisions regarding the initiation or avoidance of antibiotic therapy, particularly when uncertainty exists regarding the presence of a bacterial infection [1,17]. The strategic incorporation of PCT in these programs has demonstrated considerable potential in curbing the inadvertent and unnecessary administration of antibiotics, thereby addressing a crucial aspect of antimicrobial resistance management [1,17].

A study by Goodlet et al. found that PCT’s sensitivity for bacteremia increases with a decreasing threshold value as follows: 62% at the threshold value of 0.5 ng/mL, 76% at a threshold of 0.25 ng/mL, and 92% at a threshold of 0.1 ng/mL [18]. Meanwhile, a study by Chirouze et al. indicated that the best cutoff value for PCT was 0.4 ng/mL, which was associated with a negative predictive value of 98.8%, and a value <0.4 ng/mL accurately ruling out most diagnoses of bacteremia [19]. Hoeboer et al. claimed that a low PCT threshold could rule out the presence of bacteremia with a negative predictive value ranging from 95% to 98% [20]. PCT can also accurately discriminate between true bacteremia and false positive cultures like coagulase-negative staphylococci-contaminated blood cultures [21]. Because of its high negative predictive value, it is indeed a valuable test to rule out bacteremia.

However, we should be careful when interpreting PCT results. A study by McCraw et al. on community-acquired, post-COVID-19, MRSA pneumonia and empyema had a low procalcitonin of 0.15 ng/mL [22]. The study by Goodlet et al. showed that in over one-third of all inpatient bacteremias, PCT was not detected using the threshold of 0.5 ng/mL, and even if the strictest cutoff value of 0.1 ng/mL were applied, nearly 10% of bacteremias would still be missed [18]. *Staphylococcus aureus* was the most commonly isolated organism in patients with bacteremia and a PCT threshold of <0.5 ng/mL at 39%, of which 41% was MRSA [18]. This study showed that PCT sensitivity for bacteremia was highest for respiratory tract infection (74% at the 0.5 ng/mL threshold and 80% at the 0.25 ng/mL threshold), while it was lowest with a skin or bone infection (51% sensitivity at the 0.5 ng/mL threshold) [18]. It also showed that PCT was more sensitive to Gram-negative bacteremia than Gram-positive bacteremia (70% vs. 56%, p=0.008) [18]. A study by Jona et al. used a PCT threshold of <2 ng/mL to rule out bacteremia, a higher threshold than other studies [23]. This study showed that among patients with positive blood cultures, 20% had a negative test (PCT level <2 ng/mL), and the most common organism in this group was *Staphylococcus aureus* [23].

The high negative predictive value observed in previous studies for PCT in true bacteremia may be attributed to a low incidence of infection within the study population, leading to a greater number of true negatives compared to false negatives [18]. Interestingly, the cases discussed here with positive bacteremia caused by *Staphylococcus aureus* (a pathogen considered pathological rather than a contaminant when detected in blood cultures) had negative PCT values, even when utilizing a lower threshold of 0.05 ng/mL. This finding highlights a potential pitfall that physicians and antibiotic stewardship programs must approach with caution when interpreting PCT results. 

## Conclusions

Although PCT is not recommended to be solely relied upon in diagnosing bacterial infections, it has a high negative predictive value for ruling out bacteremia, offering valuable guidance in antibiotic stewardship programs. The rare occurrence of false-negative PCT results in cases of true *Staphylococcus aureus* bacteremia, as presented in the two discussed cases, highlight the importance of exercising caution while interpreting negative PCT results in patients with suspected bacterial infections. Physicians and antibiotic stewardship programs should be mindful of this potential limitation despite PCT's overall effectiveness as a screening tool. In the absence of an identifiable source of infection (as in the first case), sole reliance on a negative PCT as the criterion to rule out bacteremia may lead to treatment delays until blood cultures confirm the diagnosis. Further research is warranted to elucidate the specific bacterial and host factors contributing to negative PCT values in patients with true bacteremia.
